# 

**Published:** 1898-11-12

**Authors:** 


**Notices of Births, Deaths, and Marriages** are inserted in this column at a charge of 2s. 6d. for an announcement not exceeding four lines.

### NOTICE TO CORRESPONDENTS.

All MS., Letters, Books for Review, and other matters intended for the Editor should be addressed The Editor, "The Hospital," The Hospital Building, 28 & 29, Southampton Street, Strand, W.C.

The Editor cannot undertake to return rejected MS., even when accompanied by stamped directed envelope.

**Notice to Advertisers and Subscribers.**

All Advertisements, Orders for Copies of The Hospital, and Business Communications should be addressed to **THE MANAGER (Not to the Editor),** The Hospital, The Hospital Building, 28 & 29, Southampton Street, Strand, London, W.C.

The Cost of Subscription, post free, is as follows:—Per week, 2½d.; per quarter, 2s. 8d.; per half-year, 5s. 3d.; per annum, 10s. 6d. Foreign:—Per annum, 15s. 2d., payable, in all cases, in advance.

**NOTICE.—Vol. XXIV. of "The Hospital" (April, 1898, to September, 1898), in handsome cloth binding, is now on sale at the Office of this Journal, price 7s. 6d. Cases for binding the Numbers contained in this Volume 1s. 6d. Index to Vol. XXIV., 2½d., post free.**

**The Monthly Part for November is now ready. Price 6d., by post 8d.**

### BOOKS RECEIVED.

J. AND A. CHURCHILL.

"Handbook of Hygiene and Sanitary Science." By George Wilson, M.A., M.D., LL.D.Edin. Eighth edition.

THE F. A. DAVIS CO.

"Practical Uranalysis and Urinary Diagnosis." By Charles W. Purdy, M.D., LL.D.

"Primer of Psychology and Mental Disease." By C. B. Barr, M.D.

JOHN WRIGHT AND CO.

"Wright's Improved Visiting List." Compiled by Robert Simpson, L.R.C.P., L.R.C.S.

**Periodicals and Pamphlets.**—Quiver, Windsor Magazine, Scalpel, Friend of China, Woman's Signal, Medical News, Medical Record, Journal of Tropical Medicine, Literary Digest. [illegible]

### Scraps and Gleanings.

A sum of £234 has been handed to the Llanelly Hospital by Mr. Harry Studt, being the proceeds of the fête and gala held in August.

The new wing of the Sunderland Infirmary will be completed in the course of a few weeks. The nursing department will then be augmented.

The memorial stone of a new out-patient department in connection with the Stanley Hospital was laid by the Lord Mayor of Liverpool on October 13th. The new structure will cost about £2,500.

The Guardians of the Plymouth Board have appointed a committee with a view to getting a reduction made on the fees charged by the isolation hospital for the patients sent there by the Guardians.

The street collections in Cork this year in aid of Hospital Saturday amounted to £280 odd, which is an increase of £50 on last year's collection. The improved arrangements this year seem to have given great satisfaction.

The Montrose Parish Council are bestirring themselves with regard to what they allege to be a case of mismanagement at the Montrose Infirmary. There is to be a meeting next month, when the Board of the Infirmary is expected to make a statement.

The Bedford New County Hospital is rapidly approaching completion, and will be ready for occupation early next year. A further sum of £10,000 is required to enable the building to be opened free from debt, the committee having received a sum of almost £25,000, against £35,000 required.

28 & 29, SOUTHAMPTON STREET, STRAND, LONDON. W.C.

